# Valproate Treatment in an ALS Patient Carrying a c.194G**>**A Spastin Mutation and SMN2 Homozygous Deletion

**DOI:** 10.1155/2014/216094

**Published:** 2014-07-17

**Authors:** Lucio Tremolizzo, Gessica Sala, Elisa Conti, Virginia Rodriguez-Menendez, Antonella Fogli, Angela Michelucci, Paolo Simi, Silvana Penco, Christian Lunetta, Massimo Corbo, Carlo Ferrarese

**Affiliations:** ^1^Section of Neurology, Ospedale San Gerardo and DCMT, Milan Center for Neuroscience (NeuroMI), University of Milano-Bicocca, Via Pergolesi 33, 20900 Monza, Italy; ^2^Medical Genetics, A.O.U. Pisana, Ospedale Santa Chiara, Pisa, Italy; ^3^Medical Genetics, Department of Laboratory Medicine, Fondazione Serena, Ospedale Niguarda Ca' Granda, Milano, Italy; ^4^NEuroMuscular Omnicentre (NEMO), Fondazione Serena, Ospedale Niguarda Ca' Granda, Milano, Italy; ^5^Department of Neurorehabilitation Sciences, Casa Cura Policlinico, Milano, Italy

## Abstract

Here we report the case of an ALS patient found to carry both a novel heterozygous change (c.194G>A) within the spastin gene and a homozygous deletion of the SMN2 gene. The patient was started on valproic acid (VPA, 600 mg/die per os) considering the capacity of this drug of increasing survival motor neuron through an epigenetic mechanism. Patient clinical course and molecular effects of VPA on skin fibroblasts obtained from the proband are described. This c.194G>A spastin mutation might expand the previously known borders of type 4 spastic paraplegia (SPG4) and we suggest the intriguing possibility that the absence of SMN2 might have acted as a contributory risk factor for starting lower motor neuron damage. Exploring the relationship genocopy-phenocopy in selected ALS patients might represent an interesting strategy for understanding its clinical variability.

## 1. Introduction

Amyotrophic lateral sclerosis (ALS) is a neurodegenerative disorder defined by the involvement of both upper and lower motoneurons (UMN and LMN) [1]. Although several pathological mechanisms have progressively been elucidated in this disease, ALS phenotypic determinants are not completely understood, and even genome-wide association based approaches have not yet been able to penetrate such heterogeneity [2]. Considering such diversity, the possibility that genocopies and phenocopies might contribute significantly to ALS cannot be rejected and is probably still far from being completely elucidated [3].

Interestingly, ALS belongs to a clinical continuum involving other selective UMN and LMN diseases with known genetic causes, such as hereditary spastic paraparesis (HSP) and spinal muscular atrophy (SMA), respectively, offering the possibility of investigating the contribution of these factors to the clinical expression of full-blown ALS [4–6].

Here we report a very peculiar ALS case bearing both a novel missense mutation within the spastin gene (SPG4), the commonest causative gene for HSP, and at the same time the homozygous deletion of the SMN2 gene, the centromeric copy of the SMA defective gene (SMN1), known to have the potential to mitigate the clinical phenotype in SMA [7, 8] and, possibly, in ALS [9, 10], albeit in this latter case with conflicting results [11]. We anyhow hypothesized that this particular patient could be considered an unusual ALS composite genocopy, that is, expressing UMN pathology as the result of a spastin mutation, interacting with LMN pathology, which could have been predisposed by the concurrent lack of SMN2.

Aim of this paper consists not only in describing this atypical case but also in reporting the clinical and molecular effects of the treatment with valproate (VPA) that was offered to the patient considering its potential of increasing SMN levels by an epigenetic mechanism [12–14]. Moreover, in order to interpret SMN molecular data into the correct frame, the expression of this protein in skin fibroblasts obtained from our patient was compared to that assessed in a group of sporadic ALS patients and healthy controls. Interestingly, the lack of SMN2 gene copies in our patient offers the unique opportunity of selectively studying the impact of the modulation of SMN1 promoter on motoneuron disease phenotype, an issue that might be relevant when thinking that SMN1 duplications have been associated with ALS susceptibility [6] and that the loss of spliceosome integrity (related to SMN function) has been postulated to play a role in ALS [15].

## 2. Case Report

**Figure 1:**
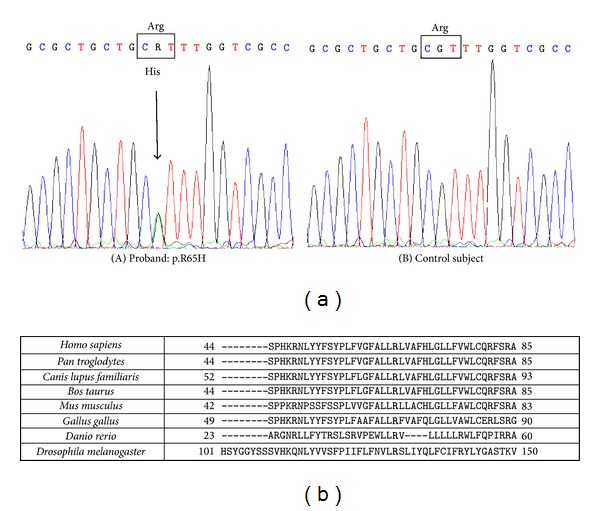
(a) Electropherograms from the proband (A) and a control subject (B). The sequence variation is a G to A transition at position 194 (GenBank Acc. no. AJ246001: c.194G>A) causing the substitution of arginine at position 65 with a histidine (p.R65H). (b) The residue at position 65 is highly conserved among different species.

A 50-year-old woman was referred in 2010 to the NEuroMuscular Omnicentre (NEMO) of Milan, Italy, due to progressively worsening limb strength deficits causing repeated falls. She complained of lower limb cramps and fasciculations associated with slowly progressing weakness since 2007. Laboratory investigations were normal. Due to the presence of clear pyramidal signs with spasticity and increased deep tendon reflexes at the lower limbs, lumbar puncture and brain and spinal cord MR scans were performed with negative results. Nerve conduction studies were normal, while needle EMG showed spontaneous activity (fibrillation potentials and positive sharp waves), increased amplitude and long duration motor unit action potentials, associated with reduced recruitment in all limbs. The bulbar district was involved as well. During a one-year follow-up period the diagnosis of ALS was established based on the clinical and neurophysiologic picture. Cognitive status was unaffected. However, family history was positive for dementia in both her father and paternal grandmother in absence of reported motor dysfunction. The proband gave her consent to specific genetic testing: SOD1, TARDPB, FUS, and C9orf72 were all negative for mutations. Due to the predominant UMN presentation, HSP-related mutations were also assessed: SPG7 was negative, while a heterozygous mutation (c.194G>A) within exon 1 of the spastin gene (SPG4) was found (Figure 1(a)). This novel SPG4 missense mutation was also found in the proband's father and her two-year-older and neurologically unremarkable sister, although it was not found in 100 healthy unrelated Italian subjects. Moreover, the residue at position 65 is highly conserved among different species (Figure 1(b)) and in silico analysis using the SIFT software (http://sift.jcvi.org/) revealed that the substitution is predicted to affect protein function with a score of 0.01 and therefore could cause loss-of-function or exert a dominant negative effect. Later on, SMN gene copies were also assessed in the proband, discovering the homozygous deletion of the SMN2 gene (exons 7 and 8).

The patient was then started on valproic acid (VPA, sodium salt) at the oral dose of 300 mg extended release b.i.d. (total 600 mg/day). Even if an initial subjective positive response was reported, the patient eventually discontinued the drug after 12 months due to the perceived lack of efficacy in contrasting disease progression. No significant changes in disease progression rates were analogously noted by the assessing physicians. No significant side effects were reported during treatment. The proband consented to skin biopsy before starting VPA treatment for fibroblast culturing. Moreover, she also consented to blood sampling both at the beginning and at the end of the period of VPA administration.

## 3. Methods

### 3.1. Fibroblast Cultures

Following ethical approval and informed consent, skin fibroblasts were obtained by biopsies performed within the forearm by a round needle (diameter 4 mm). Fibroblasts from the biopsy specimens were cultured in high-glucose Dulbecco's modified Eagle's medium containing 25 mM HEPES, supplemented with 10% heat-inactivated fetal calf serum, 100 U/mL penicillin, 100 *μ*g/mL streptomycin, and 2 mM glutamine. Cell lines were maintained at 37°C, in 95% humidified air and 5% CO_2_. Fibroblasts were always used at an equivalent number of passages of growth (ranging from 5 to 10). Culture medium was always renewed 24 h before each experiment [16].

Fibroblasts were exposed in vitro to VPA at concentrations ranging from 0.5 to 10 mM, according to the HDAC IC_50_ of this drug and to previous reports [12, 17]. Fibroblasts were at the same time obtained from 12 sporadic ALS outpatients recruited at the San Gerardo Hospital, Monza, Italy (SALS; M/F 4/8, mean age ± SD 59.5 ± 12 years, disease duration range 1–50 months, ALSFRS-R score 25.3 ± 10), and 9 age- and sex-comparable healthy controls (CTRL; M/F 4/5, age 62 ± 10 years).

### 3.2. Blood Sample Preparation

Whole-blood samples were obtained from the antecubital vein in K_3_-EDTA between 08.00 and 09.00 AM following overnight fasting. Peripheral blood mononuclear cells (PBMC) isolation was obtained by Ficoll-Histopaque density gradient centrifugation as described before [18]. PBMC pellets were then stocked at −80°C until assessment.

### 3.3. Whole-Blood Global DNA Methylation

Whole-blood global DNA methylation was evaluated by an inverse assay [19]. Briefly, 1 *μ*g of whole-blood DNA was cut O/N with a 10-fold excess of both HpaII (cutting only nonmethylated 5′-CCGG-3′ recognition sites; New England Biolabs) and MspI (cutting independently from methylation status at 5′-CCGG-3′ recognition sites; Fermentas Life Sciences) endonucleases. Each sample was then split in two aliquots incubated with 1 *μ*L of 1 : 10 diluted [3H]dCTP (60 Ci/mmol, Amersham) at 56°C for 60 min, respectively, in presence or absence of TAQ DNA polymerase (total volume 20 *μ*L). The reaction was stopped on ice and radioactivity beta-counted. Total DNA methylation was calculated as 1-(HpaII/MspI values).

### 3.4. Western Blotting

Fibroblasts were collected and homogenized in cell extraction buffer containing protease inhibitors (Sigma) and PMSF and protein concentration was assessed by Bradford's method. Serial dilutions of each sample (5, 10, 20 *μ*g) were separated by NuPAGE 4–12% Bis-Tris gels (Life Technologies) in order to ensure the subsequent readings within the linear part of the detection curve and blotted onto Hybond nitrocellulose membranes (Amersham GE Healthcare). After blocking for 1 h at room temperature (RT), samples were incubated O/N at 4°C with either anti-SMN (1 : 5,000, BD Biosciences) or anti-acetyl-histone H3 (1 : 5,000, Upstate Biotechnology) antibodies, followed by the corresponding peroxidase-conjugated secondary antibody (1 : 8,000/90 min/RT). Following ECL Plus (Amersham GE Healthcare) application, chemiluminescence was quantified by densitometer and expressed as optical density (O.D.) of the target protein and beta-actin (1 : 20,000, Sigma) used as internal standard.

### 3.5. Immunohistochemistry

Fibroblast cultures were fixed in 4% paraformaldehyde for 20 minutes and then incubated with 3% of triton (10 min) and 5% BSA (45 min) solutions. Primary antibody mouse anti-SMN (BD Transduction Laboratories) at 1 : 100 was incubated O/N at 4°C and secondary antibody Alexa Fluor 546 goat anti-mouse together with Alexa Fluor 488-conjugated phalloidin (both at 1 : 200; Invitrogen) was incubated for 1 h at room temperature. Images were taken with a confocal microscopy, Radiance 2100 microscope (Biorad Laboratories, Hercules, CA).

### 3.6. ChIP Assay

ChIP assay was performed according to the manufacturer's protocol (EZ-ChIP, Millipore). Briefly, 10e6 cells, incubated or not with increasing concentration of VPA, were treated with 1% formaldehyde. After cell lysis, cross-linked chromatin was sonicated and then immunoprecipitated with antibody against acetylated H3 (Millipore). Protein/DNA complexes were reverted; then, ChIP enriched DNA samples were quantified by real-time PCR and data expressed as percentage of input. The primer pair used to amplify SMN promoter was 5′-TTAAGGATCTGCCTTCCTTCCTGC-3′ and 5′-ATGTTGCTTAGGCCTCGTCTCGAA-3′. PCR condition was 1 cycle at 50°C for 2 min, 1 cycle at 95°C for 10 min, 40 cycles at 95°C for 15 s, 60°C for 30 s.

### 3.7. Statistical Analysis

Unpaired Student's *t*-test was used to assess the significance of differences between two treatment regimens or two subject groups, as appropriate. Correlation was computed with Pearson's *r*-test. Statistical analysis was performed by Prism 4.00 (GraphPad Software, Inc.). Data are expressed throughout the entire paper as mean ± SEM.

## 4. Results

**Figure 2:**
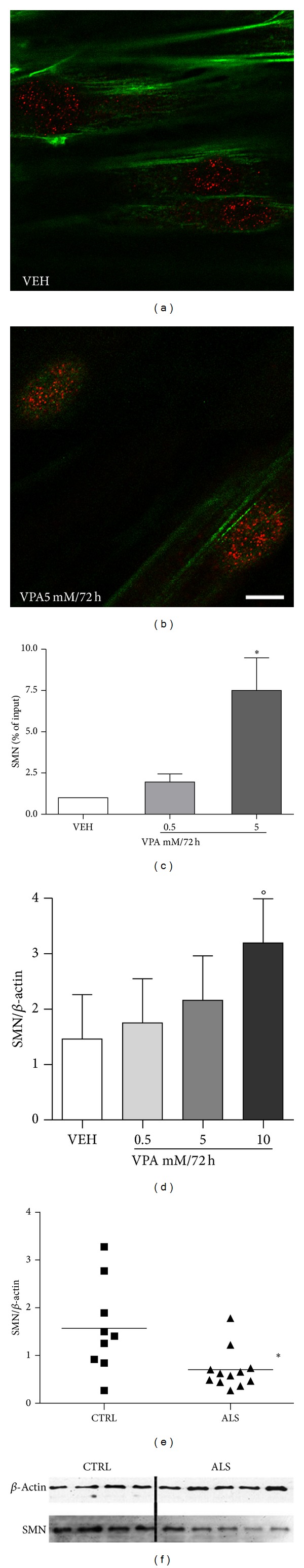
(a, b) SMN immunoreactivity in fibroblasts obtained from the proband (scale bar = 10 *μ*m): a faint (red) staining was present within nuclear structures (actin filaments counterstained in green) in vehicle-treated cells (VEH), while VPA 5 mM/72 h induced an apparent density increase of SMN-positive nuclear aggregates; (c) acetyl-histone H3 association with SMN promoter was increased in fibroblast obtained from the proband following exposure to VPA 5 mM/72 h, as semiquantified by ChIP assay (*P < 0.05); (d) SMN-like immunoreactive content in fibroblasts obtained from the proband is increased following in vitro exposure to VPA, as semiquantified by Western blotting (°*P* < 0.05); (e) (Western blotting) SMN-like immunoreactive content in fibroblasts obtained from 12 SALS patients is reduced with respect to 9 matched controls (*P = 0.01), as also shown by (f) representative immunoreactive signals (10 *μ*g of total protein) obtained in 5 different SALS patients and 4 CTRL subjects.

Fibroblasts obtained from the proband did not display major morphological alterations and a faint immunoreactive signal for SMN was present within nuclear aggregates, possibly marking nuclear gems (Figure 2(a)). VPA treatment at the concentration of 5 mM for 72 h was able to induce an apparent increase of SMN-positive nuclear aggregates with respect to vehicle-treated cells (Figure 2(b)). The same treatment was able to induce chromatin rearrangement since increased values of acetyl-histone H3 were measured (~65%, *n* = 3, data not shown) and ChIP assay documented a significantly increased association between acetyl-histone H3 and the SMN promoter (Figure 2(c)). When SMN expression was assessed following exposure to VPA at concentrations ranging from 0.5 to 10 mM for 72 h, there was a trend toward an increase of SMN-like immunoreactive signal in total cell lysates that became significant only at the concentration of 10 mM (about twofold increase, see Figure 2(d)). However, the semiquantification of basal SMN-like immunoreactive content (with respect to beta-actin) in the fibroblasts obtained from the proband was similar to that obtained in healthy controls (Figures 2(e) and 2(f)). On the other hand, SALS patients (*n* = 12) showed about 50% lower SMN-like immunoreactive content with respect to the control group (*n* = 9, Figures 2(c) and 2(f)), without any significant relationship with demographic or clinical variables. Finally, global DNA methylation in PBMC obtained before and after the period of VPA administration (12 months at the dose of 300 mg extended release b.i.d. per os) documented a decrease of ~50% of methyl-cytosine content, consistent with the demethylating properties of the drug, while SMN-like immunoreactivity was apparently unchanged (*n* = 3, data not shown).

## 5. Discussion

The c.194G>A spastin mutation apparently expands the previously known borders of type 4 spastic paraplegia (SPG4) [20], causing the substitution within exon 1 of Arg with a His at position 65 (p.R65H), highly conserved across different species as shown by in silico analysis. Arguing against the hypothesis that this genetic variation might be pathogenic, there is the lack of segregation with the neuromuscular disease within the familiar group. Furthermore, current lack of functional assays certainly does not allow establishing a conclusion about the potential pathogenic effects of the reported change. Intriguingly, however, the heterozygous duplication c.304_309dupGCCTCG within the spastin gene was previously reported in a patient affected by a very slowly progressing form of ALS [21]. The same authors further reported a case of rapidly progressing ALS bearing a heterozygous missense change (S44L) within the same gene [5]. We may conclude hypothesizing that all these spastin variations might have increased the risk for developing ALS although this hypothesis cannot be verified at the moment. Lower motoneuron involvement has already been sporadically described in similar patients (e.g., SPG17, see [22]), and we find intriguing the possibility that the absence of SMN2 might have acted as a further contributory risk factor, in our hypothesis synergistically spreading motor neuron damage. In fact, the ultimate phenotypic expression of the proband was clinically indistinguishable from that of several typical ALS cases, although initially the preponderance of upper motor neuron dysfunction prompted looking for further non-ALS specific genetic characterization. Certainly, SMN2 deletion involves a limited albeit defined quote of the population (about 8-9% in [11]) and its significance is not clarified, since it has been proposed either as a protective factor [11], a noninfluent factor [23, 24], or as a risk factor for developing ALS [9, 25].

We anyhow hypothesized that this particular patient could be considered an ALS composite genocopy, that is, expressing UMN pathology due to this novel spastin mutation that interacted with a LMN pathology, whose risk of appearance could have been increased by a concurrent minor decrease in SMN levels. Certainly, this is a speculation that cannot be directly verified, but exploring the relationship between genotype and phenotype in selected ALS patients, including different genocopies and phenocopies, might represent an interesting strategy for understanding the clinical variability strongly characterizing this disorder. Even if the exact clinical expression of this novel SPG4 mutation is not yet known, altered SMN expression has already been hypothesized to play a role in ALS with quite conflicting results [6, 9, 11], starting from the original hypothesis that the lack of this gene product might induce the same well-known dysfunction that determines and modulates SMA clinical phenotype [7, 8]. Consistent with the accessory role characterizing SMN2, SMN total levels were not significantly decreased in our patient fibroblasts. On the other hand, our SALS patients displayed a significant decrease of this protein, despite the limited number of recruited subjects, as already suggested [9]. Further studies addressing SMN levels in peripheral cells from ALS patients with respect to their specific SMN1 and SMN2 genotype might certainly clarify this issue [26].

VPA was offered to our patient in an open label course considering the epigenetic properties of this drug in different neuropsychiatric condition models and cell types [16, 27]. The systemic administration of VPA is in fact able to increase SNC levels of acetyl-histone H3 [28] and to prevent the increase in 5-methyl-cytosine content following the administration of hypermethylating agents [29]. Furthermore, SMN levels are selectively increased by the administration of VPA modifying SMN1/2 promoter structure [12, 14]; VPA has already been tested on SMA and ALS patients [30–32], albeit mainly with negative results. As a matter of fact, our patient's absence of clinical response to VPA parallels the findings of these trials [30–33] and might also be consistent with the apparently high-enough residual levels of SMN in our patient, although alternative biological explanations might apply [34]. For example, the proposed VPA dose was of 600 mg/die, conceivably corresponding to a plasma concentration ranging from sub- to just above the lower border of the recommended therapeutic window for epilepsy, plausibly close to a value roughly equivalent to the IC_50_ of this HDAC inhibitor (~0.4 mM) [17]. This dose could have been producing a quite small effect not eventually reaching clinical threshold. For this reason we decided to include in ex vivo experiments a dose one order of magnitude higher, in order to magnify the expected outcomes [12].

However, recent suggestions that modifications of the epigenome might be of interest for understanding clinical variability in ALS [19, 35] might further justify the attempts of testing in the future other compounds able to modify histone acetylation or histone and/or DNA methylation [36]. In fact, in spite of the fact that ALS clinical heterogeneity might imply differences in the involved pathophysiology, a common downstream pathway converging on the regulation of mRNA expression has been repeatedly demonstrated since the discovery of TARDP43 and FUS/TLS mutations in ALS [37, 38]. Moreover, an epigenetic dysfunction has been hypothesized in several complex disorders for which one single clear-cut mechanism cannot be demonstrated [39], such as in the case of ALS. Interestingly, TDP-43 and FUS/TLS localize to nuclear gems through an association with SMN [15, 40], implying that defective integrity of the spliceosome might be started by an expression or functional defect in any of these gene products leading, at least, to LMN dysfunction.

## 6. Conclusion

The case we report here is carrier of both a novel spastin mutation and, plausibly, a minor defective SMN production, possibly leading to a composite ALS genocopy. Describing peculiar ALS genocopies and phenocopies might be of interest for the field since dissecting the roads of phenotypic complexity in motor neuron disorders conceivably represents one way to eventually find an effective cure for ALS.
